# Pediatric Primary Care Physicians’ Perceptions of, and Processes for, Pediatric Blood Pressure Screening, Follow-Up, and Hypertension Management

**DOI:** 10.3390/children12020185

**Published:** 2025-02-04

**Authors:** Melissa Goulding, Grace W. Ryan, Elise M. Stevens, Sharina Person, Robert Goldberg, Arvin Garg, Stephenie C. Lemon

**Affiliations:** 1 Division of Preventive and Behavioral Medicine, Department of Population and Quantitative Health Sciences, University of Massachusetts Chan Medical School, Worcester, MA 01655, USA; 2 Child Health Equity Center, Department of Pediatrics, University of Massachusetts Chan Medical School, UMass Memorial Children’s Medical Center, Worcester, MA 01655, USA; 3 Division of Biostatistics and Health Services Research, Department of Population and Quantitative Health Sciences, University of Massachusetts Chan Medical School, Worcester, MA 01655, USA; 4 Division of Epidemiology, Department of Population and Quantitative Health Sciences, University of Massachusetts Chan Medical School, Worcester, MA 01655, USA

**Keywords:** blood pressure, pediatric hypertension, screening, primary care, clinical practice guidelines, qualitative

## Abstract

**Background/Objectives**: Pediatric hypertension is an important and impactful condition. The 2017 American Academy of Pediatrics clinical practice guidelines provide recommendations for identifying and managing this condition within primary care. However, the perspectives and self-described practices of physicians are largely absent in the current evidence base. We aim to fill this gap through our qualitative investigation of physicians’ perceptions and practices related to the screening, follow-up, and management of primary pediatric hypertension. **Methods**: We conducted semi-structured interviews with pediatric and family medicine physicians from the largest healthcare system in central Massachusetts. The interviews explored physicians’ perceptions, and practices related specifically to pediatric blood pressure screening, follow-up for high blood pressures, and management of primary hypertension. We used rapid qualitative analysis to synthesize data into the resulting themes. **Results**: Eleven interviews were conducted. Resulting themes included: (1) physicians are generally concerned about pediatric hypertension and familiar with guidelines, but other concerns often take precedence, (2) blood pressure screening occurs mainly during yearly well visits, (3) physicians do not trust high blood pressure readings, (4) follow-up after high blood pressure readings varies, and (5) primary care physicians typically refer to specialists for hypertension management. **Conclusions**: This study expands current literature by providing salient context to the state of pediatric blood pressure screening, follow-up, and primary hypertension management after the 2017 guidelines among primary care physicians affiliated with an academic medical center. Our findings related to physicians’ trust in electronic health record flags and the utility of follow-up by school nurses warrant further investigation.

## 1. Introduction

Mounting evidence about the impact of elevated blood pressure in children and adolescents on cardiovascular health trajectories across the life course has made the need for prevention and early detection of pediatric hypertension clear [1,2]. The American Academy of Pediatrics’ (AAP) 2017 Clinical Practice Guidelines for Screening and Management of High Blood Pressure in Children and Adolescents (CPG) call for regular blood pressure screening and follow-up after high readings in all children aged 3–17 years [2]. Recent investigations, using electronic health record data, however, indicate that adherence to these guidelines and specifically to the screening and follow-up recommendations have been suboptimal [3,4,5,6].

Efforts to improve adherence to the 2017 CPG are warranted and should be informed by the physicians who screen for, diagnose, and manage pediatric hypertension in primary care settings. Physicians’ voices and perspectives, however, have been infrequently reported in the literature. To our knowledge, investigations into physicians’ perceptions to help contextualize barriers to identifying and managing pediatric hypertension have been limited to three studies. Although these studies identified several barriers related to clinical workflow and resources, as well as physicians’ self-efficacy regarding the management of pediatric hypertension, knowledge gaps remain [7,8,9]. First, two of these studies were set in community health centers, and therefore there remains a gap in knowledge related to perceptions of physicians who practice in other settings, including academic medical centers [7,8]. Second, only two of these studies were conducted after the release of the 2017 CPG [8,9]. In the 2017 CPG, several changes were made to prior recommendations. Notably, the frequency at which screening is recommended was increased from at least once yearly to at every healthcare encounter for children who are experiencing obesity [2]. Of the recent (post-2017) studies, one was quantitative in the form of a survey, and the other focused specifically on clinics in which follow-up after a hypertension diagnosis was low and included various clinic staff [8,9]. Therefore, there remains a need to qualitatively explore general pediatric and family medicine physician perceptions related to the 2017 CPG.

The present study aims to fill existing gaps in the literature by providing perspectives of primary care physicians from pediatric and family medicine clinics that are associated with an academic medical center, which is also the largest healthcare system in Central Massachusetts, after the release of the 2017 CPG. In particular, we aim to describe physicians’ perspectives towards the practices of blood pressure screening, follow-up after a high blood pressure, and the management of primary hypertension in pediatric populations. These perspectives are critical for informing strategies to improve adherence to CPGs for pediatric blood pressure screening, follow-up, and primary hypertension management.

## 2. Materials and Methods

### 2.1. Design

Primary data collection in the form of semi-structured interviews with pediatric and family medicine physicians (M.D. or D.O.) from the UMass Memorial Health System was conducted between 31 March 2023 and 11 May 2023.

### 2.2. Interview Guide

A semi-structured interview guide was developed by the study team (who have expertise in epidemiologic, qualitative, and cardiovascular research, as well as a practicing pediatrician) with guidance from a pediatric nephrologist. To develop the interview guide, we followed a systematic framework of five steps [10]. Guided by the current literature and our prior quantitative study findings, we first drafted a standardized interview guide. In line with qualitative principles, this guide was designed to be semi-structured to allow for flexibility in response to various questions and probing. The guide was designed to uncover physicians’ knowledge, perceptions, and practice related to pediatric blood pressure screening frequency, follow-up after a high blood pressure, and management of primary hypertension. Given the expansive nature of the 2017 guidelines and our project’s goals, the interview guide focused specifically on Section 4.2 BP Measurement Frequency, Section 4.3 Patient Management on the Basis of Office BP, Section 7.2 Lifestyle and Nonpharmacologic Treatment, and Section 7.3 Pharmacologic Treatment of the 2017 CPG [2]. After development, the guide was then pilot tested both internally by the research team and externally by two practicing pediatric providers. Following expert assessment by pediatric specialists outside of the research team, the guide was revised and finalized. 

### 2.3. Setting

Physicians from the UMass Memorial Health System were recruited. This system is the largest not-for-profit healthcare system in central Massachusetts and is the clinical partner to UMass Chan Medical School. The system includes a total 27 family medicine and pediatric clinics which provide primary care to children and adolescents. There were no system-wide formal efforts to implement the 2017 AAP guidelines into practice in the system prior to this study.

### 2.4. Participant Recruitment and Data Collection

Institutional emails and professional connections were used to contact and recruit pediatric and family medicine physicians from within the system. All pediatric and family medicine physicians listed on the system’s website (117 physicians) were emailed by the study team [11]. Additionally, the recruitment email was sent to all pediatric and family medicine residents by the respective program directors. Our goal was to have family medicine and pediatrics represented, as well as physicians, with varying lengths of clinical experience. Physicians were contacted by email up to four times. Eleven physicians agreed to participate, and were all interviewed. Interviews were stopped when the team determined that thematic saturation was reached. This was the point at which no new themes emerged from additional interviews. Our finding of thematic saturation after eleven interviews is in line with empirical evidence finding that 9 to 17 interviews are a sufficient sample size to reach saturation [12].

Interviews were conducted by two team members (MG and GR). All interviews were conducted via videoconferencing, audio recorded, professionally transcribed, deidentified, and checked for accuracy.

### 2.5. Analysis

To prioritize moving toward action in the form of improved blood pressure screening, follow-up, and management, we used rapid qualitative methods, which have been shown to have a high level of concordance with traditional qualitative methods, and support action-oriented research [13,14,15].

First, the final interview guide was used to create a template summary (an outline structured around the domains addressed in the interview questions). This template was then used by team members to summarize each of the completed interviews. Two team members (MG and GR) summarized the first interview separately and met to compare and ensure consensus. Each interview was subsequently summarized by a single team member (MG or GR). The summaries were then combined to create a matrix from which the domains were reviewed and synthesized into themes [16]. During the analytic process, team members met regularly to ensure consistency across interviews and summaries, as well as to discuss findings and continually assess thematic saturation [12].

### 2.6. Member Checking

The resulting themes were reviewed through a member checking process [17]. At the end of each interview, respondents were asked if they would like to participate in member checking. Those who agreed were emailed a summary of the findings and given the opportunity to review them and provide feedback on how well their perceptions and practices were represented in the summarized results. Three physicians participated in member checking and confirmed that the results accurately represented their perceptions and practices related to pediatric blood pressure screening, follow-up, and management.

### 2.7. Ethical Approval and Informed Consent

This study was approved by the UMass Chan Institutional Review Board and verbal informed consent was attained from all participants.

## 3. Results

Thematic saturation was reached through eleven interviews, which lasted an average of 20 min each. As shown in Table 1, 54% of participants were women, and most had an M.D. degree and specialized in pediatrics. The interview probes were designed to explore physicians’ perceptions towards and practices with pediatric blood pressure screening, follow-up after a high measurement, and primary hypertension management. Five primary themes emerged which describe how hypertension in children and adolescents is perceived and addressed in the primary care setting: (1) while physicians are generally concerned about pediatric hypertension and familiar with contemporary screening guidelines, other health concerns often take precedence during patient encounters, (2) blood pressure screening occurs mainly during yearly well-child visits, (3) physicians do not trust high blood pressure readings, (4) follow-up after a high blood pressure reading varies, and (5) primary care physicians typically do not manage pediatric hypertension, instead referring to specialists. Each of these major themes is described in detail below and presented alongside subthemes and representative quotations in Table 2.

### 3.1. While Physicians Are Generally Concerned About Pediatric Hypertension and Familiar with Current Screening Guidelines, Other Health Concerns Often Take Precedence During Patient Encounters

#### 3.1.1. Concern for Pediatric Hypertension

The majority of physicians perceived pediatric hypertension as a public health concern because of its long-term consequences and its increasing prevalence alongside obesity. Two physicians reported not seeing much pediatric hypertension in their practice, however, one of which still reported viewing the condition as an important public health issue.

#### 3.1.2. General Familiarity with AAP Guidelines

Physicians described being “reasonably familiar” with existing guidelines for pediatric blood pressure screening and management. All physicians agreed that the American Academy of Pediatrics was the primary source of guidelines for clinical practice related to pediatric blood pressure. A couple of physicians additionally mentioned awareness of guidance from the American Heart Association; however, none of the physicians talked about the United States Preventive Service Taskforce recommendations. Physicians described guidelines affecting their practice mostly in terms of workflow for blood pressure screening and noted knowing where to access guidelines when in need of more details. Physicians who were still in residency training also noted that they receive one lecture a year on pediatric hypertension, and described this as being beneficial for their knowledge of guidelines.

#### 3.1.3. Other Conditions Taking Precedence

Despite the general concern for pediatric hypertension and general familiarity with the AAP guidelines, physicians acknowledged that the effects of this condition are often not seen in pediatric practice, but rather are seen during adulthood. Moreover, in pediatric practice there are other more acute and pressing conditions which often take precedence during time limited health encounters. Examples given of other conditions with more pressing concerns included mental health, asthma, and obesity.

### 3.2. Blood Pressure Screening Occurs Mainly During Yearly Well-Child Visits

#### 3.2.1. Blood Pressure Is Screened at Well-Child Visits

While a minority of physicians stated that blood pressure is measured at all visits for children over the age of 3 years, most physicians reported that blood pressure is measured at all well child visits (yearly physicals) and only at some urgent/sick visits depending on the reason for the visit. Examples of urgent/sick visits in which physicians noted that blood pressure may be measured included visits related to syncope, attention deficit hyperactivity disorder, obesity, or headache. Most physicians described blood pressure measurement as part of the medical assistant’s workflow during the rooming process. Both automatic and manual methods were used to measure blood pressure.

#### 3.2.2. Physicians Scanning for Flagged Vital Signs

Most physicians reported reviewing the patient’s vital signs at every visit since, as two physicians stated, “vitals are vital”. Physicians described their attention to vitals as being limited to a “scanning” unless one or more of the values were flagged as abnormal. All physicians explained that the electronic health record flags high blood pressure measurements with red text. However, many physicians question the accuracy of this flag. Some physicians noted that, in addition to flagging the raw blood pressure measurement, the electronic health record displays the child’s age, sex, and height-adjusted blood pressure percentile.

### 3.3. Physicians Do Not Trust High Blood Pressure Readings

#### 3.3.1. Lack of Trust

Nearly all physicians expressed a lack of trust in blood pressure measurements, sharing sentiments such as they “never” trust a high reading, are “very unlikely” to trust a high reading, and are “very skeptical” of high readings. This lack of trust stemmed from beliefs that blood pressure measurements are often inaccurate (due to improper techniques such as the child having their arm in their sweatshirt during measurement, or inappropriate cuff size being used), and/or that extraneous factors often lead to isolated high measurements (child’s anxiety, “white coat hypertension”, or the child running around prior to the measurement being taken). Another factor that contributed to a lack of trust for several physicians was the belief that the electronic health record flag was too sensitive or inaccurate, and feelings of “alarm fatigue” stemming from this. One physician (family medicine) reported having trust in high readings because their clinic has performed extensive training on proper blood pressure measurement (in adult populations). Other physicians shared they would be more likely to trust a high reading if the measure was taken at the end of the visit, if the child was calm, and if the child had known risk factors for hypertension including a family history of hypertension, was born prematurely, or had obesity.

#### 3.3.2. Barriers to Accurate Blood Pressure Measurement

A lack of cuffs of various sizes being readily available in all clinic rooms was noted as a barrier to accurate blood pressure measurement. The skill of those measuring the child’s blood pressure was also noted as a barrier with one physician stating they wish their clinic had automatic blood pressure cuffs so they would not have to worry about the accuracy of manual measurements. Due to these barriers and a lack of trust in blood pressure readings, most physicians reported taking a repeat blood pressure measurement after seeing a high blood pressure reading recorded in the electronic health record. One physician suggested that changing the workflow so that medical assistants automatically retake high readings may reduce the “alarm fatigue” caused by so many flagged readings.

### 3.4. Variability of Practice After a High Blood Pressure Screening and Barriers Related to Follow-Up

If upon repeated measurement (in the same visit) the blood pressure is sustained at an abnormally high level, this triggers a response from physicians in the form of lifestyle change advice and follow-up visits, both of which were noted to have associated barriers.

#### 3.4.1. Barriers Related to Lifestyle Management

Most physicians noted that after confirming a high blood pressure finding they would discuss lifestyle modifications with the family in the hopes of enacting change to improve the child’s blood pressure. Several physicians highlighted the importance of parental buy-in for both attendance to follow-ups and enacting lifestyle changes. Some physicians reported that parents’ ears “perk up” when they hear hypertension mentioned, while other physicians reported it being harder to obtain buy-in from parents regarding hypertension because its effects are not immediately seen in childhood, and because parents have the condition themselves. Several physicians also discussed the role that social determinants of health and socioeconomic factors play in the ability of families to make lifestyle choices and wishing there were more resources and support available to help families sustain healthy lifestyle practices. Additionally, physicians noted the complexity and difficulty of addressing lifestyle changes during short clinic visits and described a lack of educational materials to give to parents regarding lifestyle management of pediatric hypertension.

#### 3.4.2. Variability in Follow-Up Practices

In addition to lifestyle advice, most physicians described planning some form of follow-up after confirming a high blood pressure reading. However, despite all the interviewees working in the same healthcare system, there was considerable variability in the modality and timing of follow-up visits. This variability seemed to stem from varying levels of trust in high measures, leading to differing levels of significance being placed on these measures. Follow-up visits were planned to re-check children’s blood pressure after a high screening finding, since high readings across multiple occasions are needed to reach a diagnosis of hypertension. The timing of this follow-up was not consistent across physicians, despite guideline recommendations. Physicians described follow-up being scheduled “in a couple months”, “in 6 months”, and “whenever is convenient”. They also described variability in where this follow-up occurs and who is conducting it. Some physicians reported follow-ups occurring in their clinic with a physician or with a nurse, while others reported giving parents the option to have the child’s school nurse take follow-up blood pressure measurements, or even gave parents the option to use an automatic blood pressure machine at home or a local pharmacy.

#### 3.4.3. Barriers Related to Follow-Up

The ability of families to return to the clinic for a follow-up visit was noted to be a barrier and offering school nurse and home/pharmacy measurement helped to overcome this barrier. The primary barrier related to follow-up, however, was communication. For each of the follow-up modalities, communication back to the physician regarding the results of the follow-up visit was noted as a barrier. In addition to the need for changes to the clinic workflow, it was suggested that this communication barrier may be partially overcome if the electronic health record alerted physicians when a child has had high readings on multiple occasions.

### 3.5. Primary Care Physicians Typically Do Not Manage Pediatric Hypertension, Instead Referring to Specialists

When a diagnosis of hypertension is made, physicians will refer children to subspecialists, namely nephrology (sometimes cardiology), for management. Physicians described making referrals to nephrology because the specialist can investigate secondary causes of hypertension, can order ambulatory blood pressure measurement, and because the primary care physicians are not comfortable managing pediatric hypertension pharmacologically. While some physicians reported that the referral process is smooth, others reported long waits due to a shortage of pediatric nephrologists in the area. The physicians noted that training and guidance on how to effectively co-manage this condition with nephrology would be beneficial to their practice, as would the ability to order ambulatory blood pressure measurement in the primary care setting.

## 4. Discussion

The present study provides salient context to the current state of pediatric blood pressure screening, follow-up after a high blood pressure, and primary hypertension management among primary care physicians. Through this work, we uncovered several barriers to primary hypertension identification and management related to trust, communication, workflows, and resources. Important additions to the current understanding of pediatric blood pressure identification and management from this work include its description of potential pitfalls of electronic health record alerts and perceived utility of working with school nurses to obtain follow-up after a high blood pressure screening.

### 4.1. Lack of Trust in Blood Pressure Screenings and Variability in Follow-Up

We found that, in line with AAP screening recommendations, children’s blood pressures are screened at well-child visits; however, increased screening frequency for children experiencing obesity was described by few providers. This finding is supported by our team’s recent investigation of electronic health record data, which found high adherence to AAP’s yearly screening recommendation, but not to the more frequent screening recommended for children who are experiencing obesity [5]. Additionally, we found that when a blood pressure screening is high, physicians are unlikely to trust it. Nearly all physicians reported a lack trust in blood pressure measures due to inaccuracy of readings, extraneous factors causing isolated high measures, and alarm fatigue from inaccurate electronic health record flags. Our findings also suggest that this lack of trust, together with a need to prioritize other conditions that are more proximal, may be leading to the highly variable and limited follow-up found by our team and others through the investigation of electronic health records [3,6].

Competing priorities and a lack of trust in the accuracy of blood pressure measurements in children were also cited as a barriers in a recent qualitative study of staff from pediatric clinics with low follow-up rates after hypertension diagnosis [9]. Our results add to the literature highlighting physicians concerns surrounding the accuracy of child’s blood pressure measurements [7,9]. Unique to our findings was physicians’ lack of trust in electronic health record alerts. Although electronic health record alerts (or alarms) have been shown to increase the recognition of high blood pressure readings, we found that when the alarm and the readings it is flagging are not trusted to be accurate, the alarm may not have the same positive effect [18].

Alarm fatigue, measurement challenges, and a lack of trust in the actual blood pressure values may adversely affect a physician’s response to a high blood pressure reading and in turn delay follow-up, especially given the limited visit time, in which physicians have to address a plethora of concerns. While physicians did describe conducting follow-up visits after finding high blood pressure readings, this follow-up lacked urgency, varied in timing and structure, and the results were often not communicated to the physicians. One physician’s description of an encounter with a child with a high blood pressure reading exemplified this, as they noted they were unaware of follow-up readings until a year later at the next well-child visit. This highlights how barriers in the follow-up process may delay the diagnosis of hypertension, and is supported by our recent investigation of electronic health records, which found that nearly 40% of children’s follow-up blood pressure measurement occurred at least 1 year after their initial high screening [6]. Making changes to foster physicians’ trust in blood pressure readings by alleviating logistical challenges (increasing cuff availability) and standardizing clinic workflows for follow-up, especially for communication after follow-up, could streamline and improve the process of diagnosing pediatric hypertension.

Another important finding related to the need for follow-up of elevated blood pressure findings was physicians’ description of using alternative methods of follow-up, including school nurse and retail pharmacy measurement. Physicians described these methods as being important to overcome barriers to returning to clinic for follow-up visits, especially in marginalized populations, who face systemic barriers to accessing healthcare. Beyond blood pressure screening and ambulatory measurement, physicians did not describe other emerging techniques, which may be used for further diagnostics [19]. More work on understanding and supporting the use of alternative methods of follow-up to make sure that all children receive necessary follow-up in a timely and accessible manner while maintaining accuracy of blood pressure measurement is needed.

### 4.2. Primary Care Management of Pediatric Hypertension

Our findings are consistent with the findings from a recent survey of 72 primary care physicians from a national network of federally qualified health centers and qualitative study of 25 clinic staff from a pediatric network [8,9]. We found that the physicians included in the present study routinely refer children to nephrology for management after reaching a diagnosis of hypertension, rather than managing it in their practice [8,9]. While specialty referral can be important to identify secondary causes of hypertension, the AAP guidelines make recommendations for the management of elevated blood pressure and stage one hypertension in primary care.

The AAP recommends lifestyle management as the first line of treatment for pediatric hypertension. However, in the present study, physicians described feeling unable to address the complexities of lifestyle changes within the clinic because of the negative impact of community- and society-level factors on lifestyle behaviors. The present study highlights the need for interventions targeting these higher levels of influence to support families’ ability to enact healthy lifestyles, especially families who are disproportionately affected by social determinants of health. If blood pressure control is not achieved through lifestyle modifications, the AAP recommends pharmacologic management. Considering physicians’ discomfort with pharmacologic management as described in the present study and in prior work, the tendency to refer to specialists is not surprising [7,8]. As such, educational efforts to support primary care physicians in the pharmacologic management of pediatric hypertension are warranted. Together, these findings highlight how physicians’ lack of confidence in their ability to support families in lifestyle management of pediatric hypertension and managing the condition pharmacologically may prevent physicians from effectively and confidently managing pediatric hypertension in primary care.

Importantly, a lack of capacity to manage pediatric hypertension in primary care may have a negative impact on health equity. The prior literature describes historical disparities in which Latino and non-Latino Black children were significantly less likely to be referred to a specialist when compared with non-Latino White children [20]. Furthermore, the relegation of this condition to specialty settings may be especially detrimental to children from disadvantaged backgrounds and underserved areas who face a plethora of barriers to obtaining specialty care. An additional visit to a specialist could present barriers and burdens such as time needed off from work, transportation to the visit, and the financial burden of additional co-pays. As such, increasing the capacity to manage this condition within primary care could positively impact health equity.

### 4.3. Physician-Identified Opportunities for Improvement

During the time of these interviews, there were no system-wide protocols of algorithms for the evaluation and management of pediatric hypertension. Participating physicians described several opportunities to improve the screening, diagnosis, and management of pediatric hypertension which may be implemented through such system-wide efforts. Physicians described needing more educational materials and available resources for lifestyle management, wanting workflows to facilitate ordering ambulatory blood pressure measurement, and wanting training on how to effectively co-manage hypertension with pediatric nephrologists. Several providers acknowledged that often the first step by the nephrologist is to order ambulatory blood pressure monitoring. Physicians in our study noted that they are interested in placing this order to speed up the diagnostic process; however, they are unaware of how to do so. The physicians’ description of these potential solutions to barriers also suggests an acknowledgement of the role that they can play in managing this condition.

### 4.4. Study Strengths and Limitations

These qualitative findings add nuanced and contemporary context regarding pediatric blood pressure screening frequency, follow-up after a high reading, and primary hypertension management in clinics associated with a large academic medical center. This work is strengthened by its qualitative nature, the inclusion of physicians specializing in both family medicine and pediatrics, as well as diversity in the length of time that the included physicians have been practicing clinically [21]. The proportion of this sample which are female and which practice family medicine is also similar to these proportions at the state level [22]. In these ways, the study sample is representative of clinical practice within large academic medical systems. However, this work is limited in that all the physicians were affiliated with one health system, and while their demographic profiles may be similar to those statewide, physicians’ experiences and perceptions may differ. The small sample size of physicians who participated in interviews, the use of a convivence sample [23], and the low participation rate of physicians from our system contribute to potential self-selection bias. Our sample may be biased toward the inclusion of physicians who are more familiar with or interested in pediatric blood pressure. Alternatively, those who chose to participate may have done so due to an interest in research and/or more time availability. Therefore, knowledge and perceptions about pediatric blood pressure from physicians in our sample may be greater than that of other providers with less interest or experience with this topic and research. This convivence sample of physicians was, however, sufficient to reach thematic saturation [2,11]. The findings of the present study may, therefore, be unique to the context of physicians within large academic-affiliated healthcare systems where physicians often have the academic time and higher exposure to research. Our findings may not be generalizable to other settings such as independent community clinics in which physicians may have less exposure to research opportunities and less time to participate in such activities.

## 5. Conclusions

Through semi-structured interviews with pediatric and family medicine physicians, we provide important context to how pediatric blood pressure screening, follow-up after a high blood pressure, and primary hypertension management occur in primary care and is perceived by primary care physicians. Our results highlight that pediatric hypertension is perceived as a public health concern for which screening generally occurs yearly. We also identified several barriers to effective follow-up and management of this condition including a lack of trust in high blood pressure readings, variability in the extent and timing of follow-up, and lack of guidance and resources for proper management. Importantly, we detail these barriers from physicians’ perspectives, as well as the opportunities they identified for overcoming these barriers to improve the timely identification and management of pediatric hypertension within the primary care setting. Clinic system level efforts may help overcome these barriers and support identification and management of pediatric hypertension. These efforts may include increased availability of blood pressure cuffs in various sizes, quality improvement efforts to support the accuracy of electronic health record alerts for blood pressure, and protocols and algorithms in the electronic health record to standardize practices around blood pressure identification and management. Additionally, educational efforts based on the 2017 CGP to keep physicians up to date with current recommendations and to increase confidence in managing pediatric hypertension should be considered.

## Figures and Tables

**Table 1:** Demographics of participant family medicine and pediatric providers (N = 11).

		Percent or Mean (SD)
Gender Identity	Female	54.5%
	Male	45.5%
Medical Degree	M.D.	81.8%
	D.O.	18.2%
Years of Clinical Practice		9.7 (8.9)
Specialty	Pediatrics	81.8%
	Family Medicine	18.2%
Estimated Time Spent in Primary Care Clinic		40.9% (26.6%)

**Table 2:** Themes and representative quotations.

Themes and Sub-Themes	Representative Quotations
**Theme 1: While physicians are generally concerned about pediatric hypertension and familiar with current screening guidelines, other concerns often take precedence during patient encounters.**	
Concern for pediatric hypertension	*“I think [pediatric hypertension] is a concern because the most often driving cause of it is obesity in the patients that we’re seeing more and more, and so, thinking about the future implications of obesity, um, on children as they progress into their adult life. Um, I think it’s important to try to mitigate the—the consequences”* *“I would say, now, it is a much larger pediatric, public health crisis, mostly related to the, like, obesity, pandemic in pediatrics”.* *“I think it is getting bigger with the increasing rates of obesity. I think we’re seeing a lot more hypertension”* *“We don’t see the end disease states in pediatrics. So, I would say right here and now for my patients, it’s not a big threat in this moment. But later on in life, it absolutely is a major health problem”.*
General familiarity with AAP guidelines	*“I don’t follow it—it to the letter of the law”.* *“I mean I can’t quote them directly to you, but I know that there are guidelines. I know that I can refer to them pretty easily”* *“I am familiar with where to find them, but I definitely don’t have them memorized”*
Other concerns taking precedence	*“If I had to choose between intervening for depression or hypertension, I’d probably go with the depression ”*
**Theme 2: Blood pressure screening occurs mainly during yearly well-child visits.**	
Blood pressure is screened at well-child visits	*“There’s nothing that specifically pops up [as reminder to measure blood pressure]. But it’s just like you get used to the workflow that every physical after the age of 3, you should get a blood pressure”* *“I would say honestly probably like 9 times out of 10 if I’m feeling like I need a blood pressure during a sick visit, the blood pressure has been obtained”*
Physicians scanning for flagged vitals	*“I think it’s [attention to blood pressure measurement] low, unless it fires off”* *“I’ll always look at [the blood pressure], that’s for sure”* *“The letters turn red. And have a little exclamation mark on it”.* *“It [blood pressure flagging] is, [built into the chart] but I’m not sure it’s always reliable. I’m not sure who, you know, it depends on what the programmer put in, so, it’ll flag an abnormal blood pressure but, I’m not sure that it’s always age appropriate”.*
**Theme 3: Physicians do not trust high pressure readings.**	
Lack of trust	*“Often, what I find in the clinic is that there’s so many other reasons that the blood pressure is [high] it’s inaccurate, it’s not a true representation, so that’s always the first thing, is that I question whether the data is real and accurate”* *“I am very skeptical and suspicious of that reading, um, because 98% of the time, it is just an inaccurate reading”* *“I usually assume that it’s high—that that was an accurate measurement. Although I—I like my next kind of thought process is like, “Well, there’s a good chance so that they were like anxious in the doctor’s office”.* *“in my mind it’s one of two things until proven otherwise usually, which is, not a well obtained blood pressure or that the blood pressure was actually high, but in my work it’s usually like the kid was just stressed out from being, you know, in a doctor’s office”* *“If there are some medical factors that make me think more and be more, heightened to say, like, “Oh, I would suspect that this patient may be more at risk for hypertension,” … that kind of make—makes me pay a little more attention”.* *“I believe that that previous blood pressure was high, but I don’t believe that that blood pressure is always that high”* *“EPIC has this alert thing. It does this alert that’s like, “This patient is hypertensive,” except, like, it’s not always super accurate for our patients…. But you get a little bit fatigued because I often just ignore it because I know that’s usually inaccurate”.* *“Oftentimes EPIC does not truly flag, like, the correct parameters for pediatric blood pressure”.*
Barriers to accurate measurement	*“If I would know they’re calm, they’re not worried, they’ve been sitting quietly for a while, the nurse is able to get a good measure, then I’m more concerned that it’s a real reading”* *“One thing that would be really helpful is, like, we often—it’s hard to find larger cuffs. We don’t—we really don’t have blood pressure cuffs in every room in our clinic”.* *“I find that the MAs will measure blood pressure, it flags red, and no one has kind of preemptively tried to repeat it”*
**Theme 4: Follow-up after a high blood pressure reading varies.**	
Barriers related to lifestyle management	*“The first thing that we do is like—like, advise them about lifestyle modification, so exercising, DASH diet, decreasing salt intake”.* *“I’ll usually start with diet, just common things, most kids will have a high blood pressure if they’re, you know, eating a lot of fast food, or really just eating out. The, I mean, the no. 1 thing that I tell parents to try and have their kids eat healthier is try and eat out as little as possible –it’s ungodly how much salt and fat every restaurant puts in their food”* *“We don’t really give them like handouts. We don’t explain like the risks of hypertension. We don’t give them like a paper that says this is DASH diet. Um, I don’t think we have those resources in my clinic. So, I think it would be useful if we have something like to be able to give out to the patients”.* *“I think that always having, like, more access to lifestyle supports is helpful, especially for kids who are from, like, lower socioeconomic backgrounds, and just have a lot less access to, like, healthy food and exercise, and just, like, parental knowledge about a healthy lifestyle. And I think that’s really hard to accomplish in a 30-min physical when you’re, also focusing on a lot of other things. I always wish there were more resources that were available to kids”*
Variability in follow-up	*“depending on how high it [blood pressure] is, if it’s like really high, I’d have them come back to the office a couple times to repeat it. But also most kids are in school, and so if possible I’ll ask the school nurse to do some readings as well so we got some readings outside the office”* *“I mark it [high blood pressure] as a concern, and then I have them come for a blood pressure follow-up, I mean, really, whenever is convenient for them. Most of them don’t come, unfortunately”*
Barriers related to follow-up	*“if a school nurse didn’t follow-up, I might not realize that until the next year”* *“Our clinic, like so many clinics, is short staffed, and so, I think just whenever that happens, the communication does get worse because people are pulled –in more different directions, um, but I think generally speaking, I think it goes okay”.* *“I’ve certainly had patients where they’ve come back for the repeat, I’ve never known – been the wiser of it, and then I see them, like, a year later for their physical, and I’m like, “Oh yeah, this came up last year at your physical. You came back two weeks later for a repeat, but now it’s, you know, a year later, and I’m getting your third measurement”* *“There can be difficulty in follow-up itself, especially because we were saying, you know, it can be hard to see, like, a clinical manifestation of high blood pressure in an otherwise healthy person”.* *“It’s just I think that unlike asthma which we mentioned like parents don’t see you know anything about blood pressure. I think it is sometimes hard to get a buy-in even when the blood pressure is high. And you know the parents will just say, “Oh, yeah, mine’s always high at the doctor’s office,” you know things like that. I think they don’t see it as much of a concern as often”.* *“That’s the nice part about pediatrics, is, like, right or wrong, I’ve found that parents are more likely to make healthy changes for their kids ahead of making healthy changes for themselves, um, but for hypertension, you kind of have to have buy-in on the parents themselves because, um, a lot of it is that kind of – of food and exercise”.*
**Theme 5: Primary care physicians typically don’t manage pediatric hypertension, instead referring to specialists.**	
	*“We don’t usually start them on antihypertensives because, like, we depend on the sub-specialty to do further workup of just like any secondary causes, and then they can manage it”* *“Usually, we send them to Nephrology. We’re not the main ones starting blood pressure medications”.* *“Most kids I’ll end up referring for hypertension treatment just because it’s more complex than adults”.* *“So, it may be helpful just to have a little education. Like hey, if your patient hasn’t been seeing a specialist …. Should I be checking labs? Should I be referring them to other specialty care?”* *“I know Nephrology a lot of times does like the 24 h blood pressure monitor. And I don’t know if you know in some ways it might be easier if like we could just—give somebody that if we were really worried”*

## Data Availability

The data are not publicly available due to technical/time limitations.

## References

[1] Falkner B., Gidding S.S., Baker-Smith C.M., Brady T.M., Flynn J.T., Malle L.M., South A.M., Tran A.H., Urbina E.M., on behalf of the American Heart Association Council on Hypertension (2023). Pediatric Primary Hypertension: An Underrecognized Condition: A Scientific Statement from the American Heart Association. Hypertension.

[2] Flynn J.T., Kaelber D.C., Baker-Smith C.M., Blowey D., Carroll A.E., Daniels S.R., De Ferranti S.D., Dionne J.M., Falkner B., Flinn S.K. (2017). Clinical Practice Guideline for Screening and Management of High Blood Pressure in Children and Adolescents. Pediatrics.

[3] Carroll A.J., Tedla Y.G., Padilla R., Jain A., Segovia E., Moin A., Wallace A.S., Sanuade O.A., Langman C.B., Mohanty N. (2023). Adherence to the 2017 Clinical Practice Guidelines for Pediatric Hypertension in Safety-Net Clinics. JAMA Netw. Open.

[4] Rea C.J., Brady T.M., Bundy D.G., Heo M., Faro E., Giuliano K., Goilav B., Kelly P., Orringer K., Tarini B.A. (2021). Pediatrician Adherence to Guidelines for Diagnosis and Management of High Blood Pressure. J. Pediatr..

[5] Goulding M., Ryan G., Frisard C., Stevens E.M., Person S., Goldberg R., Garg A., Lemon S.C. (2023). Disparities in Receipt of Guideline-adherent Blood Pressure Screening: An Observational Examination of Electronic Health Record Data from a Massachusetts Healthcare System. J. Pediatr..

[6] Goulding M., Ryan G., Frisard C., Stevens E., Person S., Goldberg R., Garg A., Lemon S.C. (2023). Pediatric High Blood Pressure Follow-Up Guideline Adherence in a Massachusetts Health Care System. Acad. Pediatr..

[7] Bello J.K., Mohanty N., Bauer V., Rittner S.S., Rao G. (2017). Pediatric Hypertension: Provider Perspectives. Glob. Pediatr. Health.

[8] Carroll A.J., Mohanty N., Wallace A.S.P., Langman C.B., Smith J.D. (2023). Perspectives of Primary Care Clinicians on the Diagnosis and Treatment of Pediatric Hypertension. Fam. Community Health.

[9] Zaidi A.H., Sood E., De Ferranti S., Gidding S., Zadokar V., Miller J., Kazak A. (2024). Parent and Primary Care Clinician Perceptions About Pediatric Hypertension. JAMA Netw. Open.

[10] Kallio H., Pietilä A.M., Johnson M., Kangasniemi M. (2016). Systematic methodological review: Developing a framework for a qualitative semi-structured interview guide. J. Adv. Nurs..

[11] UMass Memorial Health. https://www.ummhealth.org/?gclid=CjwKCAjwjMiiBhA4EiwAZe6jQ5fK-bJ1PGi9JarQB1_zx_g9_n6dMVUB47KAXiULkyC1KZzcb3KkRRoCZHAQAvD_BwE&gclsrc=aw.ds.

[12] Hennink M., Kaiser B.N. (2022). Sample sizes for saturation in qualitative research: A systematic review of empirical tests. Soc. Sci. Med..

[13] Taylor B., Henshall C., Kenyon S., Litchfield I., Greenfield S. (2018). Can rapid approaches to qualitative analysis deliver timely, valid findings to clinical leaders? A mixed methods study comparing rapid and thematic analysis. BMJ Open.

[14] Vindrola-Padros C., Johnson G.A. (2020). Rapid Techniques in Qualitative Research: A Critical Review of the Literature. Qual. Health Res..

[15] Rapid Qualitative Analysis: Updates/Developments. https://www.hsrd.research.va.gov/for_researchers/cyber_seminars/archives/video_archive.cfm?SessionID=3846.

[16] Averill J.B. (2002). Matrix Analysis as a Complementary Analytic Strategy in Qualitative Inquiry. Qual. Health Res..

[17] Slettebø T. (2021). Participant validation: Exploring a contested tool in qualitative research. Qual. Soc. Work..

[18] Brady T.M., Neu A.M., Miller E.R., Appel L.J., Siberry G.K., Solomon B.S. (2015). Real-Time Electronic Medical Record Alerts Increase High Blood Pressure Recognition in Children. Clin. Pediatr..

[19] Kaplinski M., Griffis H., Liu F., Tinker C., Laney N.C., Mendoza M., Cohen M.S., Meyers K., Natarajan S.S. (2022). Left Ventricular Measurements and Strain in Pediatric Patients Evaluated for Systemic Hypertension and the Effect of Adequate Anti-hypertensive Treatment. Pediatr. Cardiol..

[20] Flores G., Olson L., Tomany-Korman S.C. (2005). Racial and Ethnic Disparities in Early Childhood Health and Health Care. Pediatrics.

[21] Tenny S., Brannan J.M., Brannan G.D. (2024). Qualitative Study. StatPearls.

[22] Petterson S., Wilkinson E., Kessler A.C., Stone C., Bazemore A. (2018). The State of Primary Care Physician Workforce.

[23] Stratton S.J. (2021). Population Research: Convenience Sampling Strategies. Prehospital Disaster Med..

